# Proprioceptive Feedback through a Neuromorphic Muscle Spindle Model

**DOI:** 10.3389/fnins.2017.00341

**Published:** 2017-06-14

**Authors:** Lorenzo Vannucci, Egidio Falotico, Cecilia Laschi

**Affiliations:** The BioRobotics Institute, Scuola Superiore Sant'AnnaPontedera, Italy

**Keywords:** neuromorphic sensing, proprioceptive sensors, neurorobotics, neuromorphic hardware, muscle spindle

## Abstract

Connecting biologically inspired neural simulations to physical or simulated embodiments can be useful both in robotics, for the development of a new kind of bio-inspired controllers, and in neuroscience, to test detailed brain models in complete action-perception loops. The aim of this work is to develop a fully spike-based, biologically inspired mechanism for the translation of proprioceptive feedback. The translation is achieved by implementing a computational model of neural activity of type Ia and type II afferent fibers of muscle spindles, the primary source of proprioceptive information, which, in mammals is regulated through fusimotor activation and provides necessary adjustments during voluntary muscle contractions. As such, both static and dynamic γ-motoneurons activities are taken into account in the proposed model. Information from the actual proprioceptive sensors (i.e., motor encoders) is then used to simulate the spindle contraction and relaxation, and therefore drive the neural activity. To assess the feasibility of this approach, the model is implemented on the NEST spiking neural network simulator and on the SpiNNaker neuromorphic hardware platform and tested on simulated and physical robotic platforms. The results demonstrate that the model can be used in both simulated and real-time robotic applications to translate encoder values into a biologically plausible neural activity. Thus, this model provides a completely spike-based building block, suitable for neuromorphic platforms, that will enable the development of sensory-motor closed loops which could include neural simulations of areas of the central nervous system or of low-level reflexes.

## 1. Introduction

In recent years, the development of action-perception closed loops that include biologically inspired neural network has risen at a rapid pace (Knoll and Gewaltig, 2016). These closed loops can be useful in both robotics and neuroscience. From a robotic perspective, these neural networks contribute to the creation of a new class of robotic controllers that could be capable of dealing with the increasing complexity of physical systems, some of which are built with hardware compliance (Negrello et al., 2015) or muscle-like actuation mechanisms (Nakanishi et al., 2012). On the neuroscientific side, such loops could provide the necessary input/output connections, in a rich environment, for detailed, full-scale neural simulations that model a specific part of the nervous system, such as the cerebral cortex (Potjans and Diesmann, 2014). However, to close these loops, it is crucial to find ways to connect physical or simulated embodiments (i.e., robots or musculoskeletal systems) to these networks that mimic neural behaviors. In particular, one must find ways to translate sensory information into neural activity (i.e., spikes or synaptic currents) to connect sensors to brain model simulations, providing a spike-based or neuromorphic translation. In most animals, the brain receives and integrates information from different sensory pathways: proprioceptive, exteroceptive, and introceptive ones. In particular, proprioceptive feedback is crucial when performing voluntary movements, and its malfunction can possibly produce severe impairments such as dystonia (Kaji et al., 1995). Therefore, in the context of developing closed loops, it is necessary to properly translate proprioceptive sensory information gathered from the embodiments into a neural activity suitable for the brain model employed.

For physical robotic applications, it is suitable to embed hardware neuromorphic sensors that can natively transmit information as spike trains. Such solutions have been proved effective to translate visual information, for example by employing dynamic vision sensors to learn how to perform obstacle avoidance (Stewart et al., 2016) or to give input to vision systems integrating different eye movements (Mulas et al., 2015; Vasco et al., 2016) and to process auditory stimuli (Gomez-Rodriguez et al., 2007; Chan et al., 2012). However, no hardware mechanism for event-based proprioceptive feedback has been developed.

Several software solutions for generating spiking activity from proprioceptive information in closed loops have been developed. They can fall into one of two categories depending on how the translation is achieved: custom or biologically inspired ones. The first approach usually relies on tailor-made translations, specific for the tasks to be solved. In Bouganis and Shanahan (2010), proprioceptive feedback from the encoders of iCub arm joints is translated in the firing rate of a population of neurons by dividing the range of the joints into bins and assigning a firing rate normally distributed around the neuron encoding the current joint angle. Casellato and colleagues converted visual and sensory information on a state and an error signal that were then translated into a firing activity through radial basis functions for the control of a robotic arm, using a spiking network embedding a cerebellar model that included a learning mechanism (Casellato et al., 2014). A similar model was also used to control a single joint of a musculoskeletal robot (Richter et al., 2016). Folgheraiter and Gini presented a model of low-level reflexes for a tendon-driven hand in which analog values for the sensors are translated into firing rates in a proportional fashion (Folgheraiter and Gini, 2004).

In contrast, biologically inspired translation approaches are developed by implementing simulations of sensory processing mechanisms found in animals, and are therefore more suitable for sending spiking activity to more realistic models of the nervous system. However, very few examples of this type exist in the current state of the art. Among these, a simple model of the muscle spindle activity has been employed to perform this translation in a closed loop between a musculoskeletal simulation and a neural model replicating low-level reflexes (Sreenivasa et al., 2016). A more complex model of the muscle spindle, albeit not completely spike-based, has been used to close the loop with a cadaver finger to create a neuromechanical system (Niu et al., 2017) and with a musculoskeletal simulation to study control of the human posture (Elias et al., 2014).

To provide a fully spike-based, biologically inspired translation model, we relied on insights from biology and neuroscience. In mammals, proprioceptive information is transmitted to the central nervous system from the Golgi tendon and muscle spindle organs. In particular, the muscle spindles are the main source of proprioceptive feedback for spinal sensorimotor regulation and servocontrol. This specialized type of fiber is found inside muscles, lying along extrafusal fibers, and provide information about the length and velocity of the muscle. Several models of afferent activity coming from muscle spindles have been developed over the years. Many of these model the firing rate of the afferent fibers as a polynomial function of the muscle stretch and stretch speed (Matthews and Stein, 1969; Chen and Poppele, 1978; Houk et al., 1981; Hasan, 1983; Prochazka and Gorassini, 1998). These works can be classified in three groups: models based on linear transfer functions (Matthews and Stein, 1969; Chen and Poppele, 1978), models based on curve fitting relying on non-linear transfer functions (Houk et al., 1981; Hasan, 1983), and non-linear models relying on biological evidence of the muscle spindle (Otten et al., 1995). An interesting comparison of the firing prediction of these models, evaluated according to the hamstring spindle primary afferent firing recorded during normal stepping in cats, has been proposed by Prochazka and Gorassini (1998). The authors include in this work also a new hybrid model able to fit neurophysiological data more closely. All the mentioned works, albeit efficient and easy to implement, are incomplete, as they lack two important features: response to fusimotor stimulation and distinction between primary and secondary afferent activity. In particular, fusimotor stimulation from γ-motoneurons changes the spindle's relative sensitivities over the wide range of lengths and velocities that occur during different tasks (Banks, 1994). Neuroscientific evidence indicates that this could be used in detecting changes in the desired trajectory of the intended movements, such as in locomotion (Ellaway et al., 2015). For these reasons, recently, more complex spindle models that include fusimotor modulation of the afferent responses have been developed (Lin and Crago, 2002; Maltenfort and Burke, 2003; Mileusnic et al., 2006). In the work proposed in Maltenfort and Burke (2003), the authors developed a computational model of the primary afferent activity considering the response to combinations of stretching during mixed dynamic and static fusimotor effects without considering secondary afferent activity. In Lin and Crago (2002), the model is more comprehensive, considering primary and secondary activities, but because of the high number of free parameters that must be tuned, it is not suitable to be integrated on different embodiments.

In this work we propose a completely spike-based, biologically inspired mechanism for the translation of proprioceptive feedback that implements a computational model of muscle spindle activity. In particular, the proposed model is based on Mileusnic et al. (2006), as it is shown to be complete, including fusimotor activation and primary and secondary afferent activities, and is suitable for an implementation in closed loops (Niu et al., 2017). In principle, such a model should be used in conjunction with a detailed spiking simulation of neural pathways descending from the central nervous system that include γ-motoneuron activations. However, the original model is completely rate based. Therefore, we extended it to cope with such a spiking input, and we included spike trains generation for the output of the model, developing a fully spike-based system. To provide a solid building block that could be used in both simulated environments and real-time scenarios, we decided to integrate it in a spiking simulation and to implement it for a commonly used spiking neural simulator, NEST (Gewaltig and Diesmann, 2007), and for a neuromorphic hardware platform, SpiNNaker (Khan et al., 2008). To prove the correctness of the two implementations, a validation and a comparison have been performed. Finally, to prove the effectiveness of the proposed model in translating sensory information, experiments in which it has been coupled with simulated or physical systems are presented.

## 2. Materials and methods

### 2.1. Muscle spindle model

**Figure 1:**
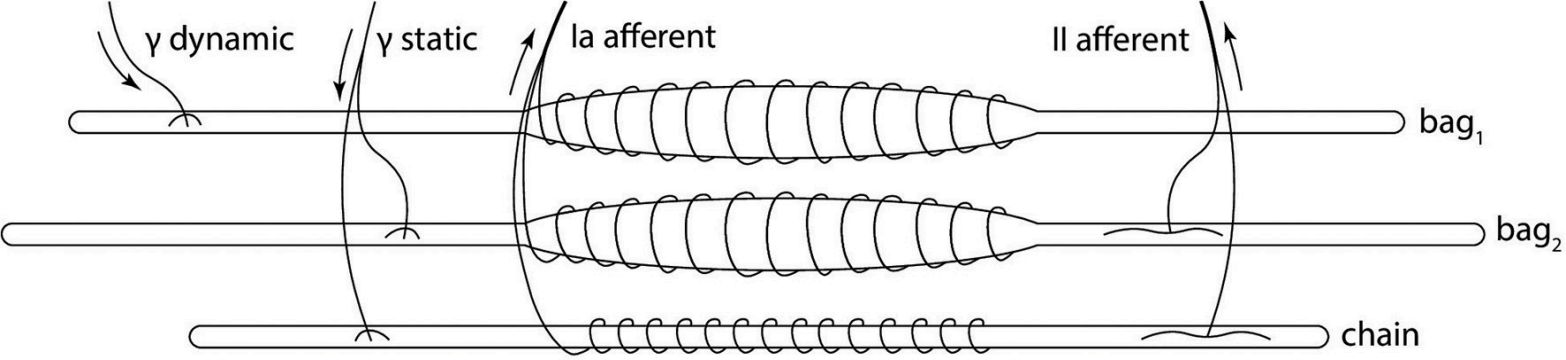
Biological model of the muscle spindle. The three intrafusal fiber types receive different fusimotor stimulations (static and dynamic) and produce primary (Ia) and secondary (II) afferent activities. Adapted from Proske (1997).

The muscle spindle organ consists of three types of intrafusal fibers: bag_1_ and bag_2_, which are longer and nuclear, and the shorter chain type (Boyd, 1981). These types of fibers react differently to the two different types of fusimotor activations, and their activity is combined to produce primary and secondary afferent activity (Figure 1). Activity of dynamic γ-motoneurons only affects bag_1_ fibers, while the sensitivity of bag_2_ and chain fibers is regulated by static γ-motoneurons. The firing rate of primary afferent (Ia) is a combination of all the fiber activity, while only bag_2_ and chain fibers contribute to the secondary afferent (II) rate. Because of the anatomy, Ia afferent endings carry information to the central nervous system that depends on both the length and stretch speed of the muscle, while II afferent endings provide information relative mostly to the length.

In Mileusnic et al. (2006), the authors propose modeling all intrafusal fiber types with the same function, with different parameters based on physiology. This functions have two inputs: the fascicle length *L* (and its derivatives $\dot{L},\ddot{L}$) and the relevant fusimotor activation level (*f*_*dynamic*_, *f*_*static*_). The fiber model consists of a sensory (*SR*) and polar (*PR*) regions, modeled as a pure elastic element and as a spring with a parallel active contractile element, respectively. From these models, given the inputs, one can compute the tension of the whole fiber *T* through a second-order differential equation. Once the tension is computed, the contribution of the fibers to the afferent rates can be obtained as follows:

(1) $$rate_{bag_{1}}(t)=G\cdot \left[\frac{T(t)}{K^{SR}}-(L_{N}^{SR}-L_{0}^{SR})\right]$$

(2) $$rate_{bag_{2}}(t),rate_{chain}(t)=G\cdot \left\{\begin{array}{l} X\cdot \frac{L_{secondary}}{L_{0}^{SR}}\cdot \left[\frac{T(t)}{K^{SR}}-(L_{N}^{SR}-L_{0}^{SR})\right]+\\ +(1-X)\frac{L_{secondary}}{L_{0}^{SR}}\left(L(t)-\frac{T(t)}{K^{SR}}-L_{0}^{SR}-L_{N}^{PR}\right) \end{array}\right\}$$

A complete description of all the parameters found in Equations (1)–(8) and their values, which differ for every type of fiber, can be found in Mileusnic et al. (2006), Table 1. For a more detailed discussion of how the fibers are modeled, refer to Mileusnic et al. (2006), from which Equations (1)–(4) and (6) and (7) were adapted. The contributions are then combined to generate the firing rates of primary and secondary afferents:

(3) $$rate_{II}(t)=rate_{bag_{2}}(t)+rate_{chain}(t)$$

(4) $$rate_{Ia}(t)=\{\begin{array}{ll} rate_{bag_{1}}(t)+S\cdot rate_{II}(t) & \text{if }rate_{bag_{1}}(t)>rate_{II}(t)\\ rate_{II}(t)+S\cdot rate_{bag_{1}}(t) & \text{if }rate_{bag_{1}}(t)<rate_{II}(t) \end{array}$$

therefore computing the secondary afferent rate as the direct sum of bag_2_ and chain activities, and the primary as a weighted sum between the three, which models the partial occlusion effect described in neuroscientific experiments. Such rates can be used to compute inter-spike time intervals to generate discrete spike events in a spiking neural simulator.

Because of the computation of *T*(*t*) as a second-order differential equation, the original model is not well suited for the integration in applications where information regarding acceleration is unavailable or very noisy. This is because even a medium level of noise can lead to instability while performing a double integration. To avoid this, we decided to simplify the model by setting $\ddot{L}=0$. The impact of this change is small, as the mean difference between the afferent firing rates with and without acceleration, computed on a Simulink implementation of the original model, was lower than 1% in most cases. This simplifies the tension equation for the polar and sensory regions, which can now be rewritten as follows:

(5) $$T(t)=\{\begin{array}{ll} K^{SR}\cdot \left(L(t)-L^{PR}(t)-L_{0}^{SR}\right) & \text{for sensory region}\\ \begin{array}{l} \beta (t)\cdot C\cdot \left(L^{PR}(t)-R\right)\cdot \\ \text{sign}\left(\dot{L^{PR}(t)}\right)\cdot {\left|\dot{L^{PR}(t)}\right|}^{a}+\\ K^{PR}\cdot \left(L^{PR}(t)-L_{0}^{PR}\right)+\Gamma (t) \end{array} & \text{for polar region} \end{array}$$

where β and Γ depend on the current fusimotor activation:

(6) $$\beta (t)=\beta_{0}+\beta_{1}\cdot f_{dynamic}(t)+\beta_{2}\cdot f_{static}(t)$$

(7) $$\Gamma (t)=\Gamma_{1}\cdot f_{dynamic}(t)+\Gamma_{2}\cdot f_{static}(t)$$

Given that the tension of the two region of the fiber is the same, Equation (5) can be rearranged into a first-order differential equation:

(8) $$\dot{T}(t)=\dot{L}(t)-signpow\left(\frac{T(t)-K^{PR}\cdot \left(L(t)-L_{0}^{SR}-\frac{T(t)}{K^{SR}}-L_{0}^{PR}\right)-\Gamma (t)}{\beta (t)\cdot C\cdot \left(L(t)-L_{0}^{SR}-\frac{T(t)}{K^{SR}}-R\right)},a\right)\cdot K^{SR}$$

where

(9) $$signpow(x,a)=\text{sign}(x)\cdot |x{|}^{a}$$

After this rearrangement we have a model, Equation (8), whose only inputs are $L,\dot{L},f_{dynamic}$, and *f*_*static*_. Information coming from actual sensors can thus be processed to generate *L* and $\dot{L}$, in units of the rest fascicle length *L*_0_, to provide dynamic input to the model.

### 2.2. Spiking fusimotor input and parameter identification

In Mileusnic et al. (2006), *f*_*dynamic*_ and *f*_*static*_ activations with values between 0 and 1 are computed from the actual fusimotor frequencies γ_*dynamic*_ and γ_*static*_ using a biochemical Hill-type equation from analog values of the firing rates, in conjunction with low-pass filtering. However, this solution relies on the instantaneous firing rates of γ-motoneurons. Such rate, in a spiking neural network simulation, cannot be accurately computed without introducing delays, as using the inter-spike interval can lead to very noisy results in case of irregular spike trains, while averaging it over time bins would make the rate smoother but it would introduce delays. Therefore, this type of computation is not suitable to be integrated in a spiking neural network that simulates the activity of γ-motoneurons. In fact, the spindle model should receive spike events and integrate them to compute the activation levels of fusimotor activity. To do so, we decided to employ a spike integration mechanism, adding spikes with an instantaneous response and exponential decay, similarly to an exponential synapse model. The actual spike response *r* was scaled with respect to the current value of the activation *f* to keep the results between 0 and 1:

(10) $$\dot{f}(t)=\left(e^{-\frac{1}{\tau }}-1\right)\cdot f(t)+r\cdot (1-f(t))\cdot u(t)$$

(11) $$u(t)=\{\begin{array}{ll} 1 & \text{if a spike is received at time }t\\ 0 & \text{if no spikes are received at time }t \end{array}$$

where *r* is the maximum impulse response and τ is the decay time. In the reference model, the activation level for chain fibers was not implemented with a low-pass filter but as a direct saturation function of the static γ-motoneuron firing rate. However, as already stated, it is not possible to compute the instantaneous firing rate during spiking network simulations, so we decided to employ the same spike integration technique for the fusimotor activation of chain fibers. Another possible approach to spike integration is to employ α-shaped responses. This leads to similar results; however, such a spike integration technique cannot be implemented with the SpiNNaker APIs, as they currently only support exponential synapses.

We then employed a model selection procedure to select the maximum impulse response *r* and decay time τ for the spike integration mechanism (Equation 10). In the original model, the activation level of fusimotor activation under constant γ-motoneurons activity rose and stabilized at a certain maximum value. Therefore, we created a dataset of these maximum values, using the Hill-type activations, for different frequencies of motoneurons firing rates: {10, 50, 75, 100, 150}spikes/s. Then we performed a model selection procedure on all the different combinations of values for *r* and τ ranging in 0.01 ≤ *r* ≤ 0.4 and in 100 ms ≤ τ ≤ 500 ms, with discrete sampling. To evaluate the parameters, we simulated the spike integration at a given frequency for 3 s, and we computed the percentage error of the obtained maximum activation levels compared with those in the dataset (*magnitude error*). As it reported in Mileusnic et al. (2006), fusimotor activation reaches 90% of the maximum value in 343 ms for *f*_*dynamic*_ and in 471 ms for *f*_*static*_. To match this property, we also evaluated the obtained percentage at these specific points in time and computed the discrepancies with 90% (*shape error*). During the parameter identification procedure, input for the spike integration was simulated by generating spikes at fixed intervals, which produced a regular oscillation in the computed activations. To limit the effect of these oscillations on the selection procedure, errors were computed on a smoothed activation. For each parameter combination, we summed up the magnitude and shape errors, and we averaged them on the different frequencies of the dataset. At the end of the procedure, we found that these values had the smallest average percentage error (7%):

$$\begin{array}{l} r_{dynamic}=0.08,\;\tau_{dynamic}=310\text{ms}\\ \;r_{static}=0.09,\;\tau_{static}=425\text{ms} \end{array}$$

**Figure 2:**
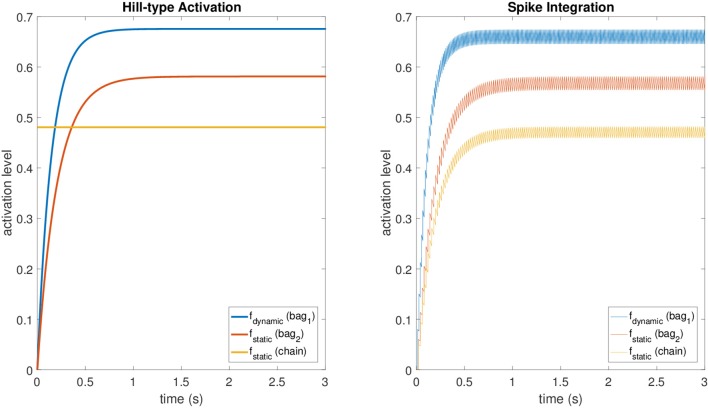
A comparison between activation levels obtained with the Hill-type equations (with low pass filtering) and those obtained with the spike integration procedure for a constant dynamic fusimotor stimulation of 75 spikes/s and a static stimulation of 50 spikes/s. The regular oscillation of the activation levels in the spike integration case is produced by the input spike trains, that were generated at fixed time intervals.

These values provide accurate results for the fusimotor activity ranging from 30 spikes/s to 150 spikes/s, while they tend to make the spike integration mechanism underestimate *f* for lower stimulation frequencies and to overestimate it for higher ones. For the static activation level of the chain fiber, we scaled *f*_*static*_, relative to bag_2_, by a factor equal to the mean scaling factor between the maximum levels of the original model at the same stimulation frequencies of the dataset. The averaged scaling factor was 0.829. A comparison between the original activations and those obtained with the spike integration procedure can be seen in Figure 2, where constant dynamic and static fusimotor activities (γ_*dynamic*_ = 75 spikes/s and γ_*static*_ = 50 spikes/s) are converted into the corresponding fusimotor activation levels.

### 2.3. Implementation

To create a robust, reliable component, we decided to integrate into two commonly available simulation platforms: NEST and SpiNNaker. NEST is a point-neuron simulator for spiking neural networks with an extendible comprehensive library of different neuron and synapse models, including plasticity mechanisms. It focuses on the accuracy and scalability of the simulation, and it can run networks of any size and topology, possibly exploiting supercomputing but with no simulation time constraints. SpiNNaker, however, offers a hardware platform with a massively parallel set of low-energy cores, and it focuses on real-time simulations. Currently, the library of neural models and synapses is limited, but the provided APIs ease the development of new ones. In our experiments, we employed a SpiNN-5 board with 48 chips, each one with 18 cores.

For NEST, the implementation proceeded by using the provided APIs to create a new neural model that can be simulated alongside other neuron types and receive spike events. The fiber tension for each type of fiber was computed by performing a discrete fixed-step integration of Equation (8). Fusimotor activation was computed by the aforementioned spike integration (Equation 10). Spikes are transmitted to the spindle model through two different synapse types, representing dynamic and static efferent fibers, to separate the incoming spike events. After the computation of the Ia and II afferent rates, actual spikes are generated using a Poisson distribution for the inter-spike interval, employing the existing NEST utilities. Because in NEST simulation every neuron model can have only one output channel, a single spindle unit cannot produce both primary and secondary afferent activities. To overcome this limitation, we added a Boolean flag to the model that can be used to switch between the two afferent types, defaulting to primary. As a consequence, if one wants to simulate a certain number *n* of complete muscle spindles, he should create 2*n* units and set the appropriate flags for half of population. Another possibility, equivalent in terms of produced output and performances, would have been to develop two separate models for primary and secondary activity. However, we decided to pursue the first strategy to provide a uniform interface with the SpiNNaker model, where the two choices are not equivalent, as described in the next paragraph. At the end of the implementation, the model was available both from the SLI interface and from PyNEST, and its parameters, such as the fiber length and speed, can be set using standard NEST calls. However, as with any other NEST model parameter, to set it to a new value, the simulation must be stopped and resumed after the change.

The same procedure was employed for the implementation on the SpiNNaker neuromorphic hardware, using a fixed-step integration of Equation 8 and spike integration for the fusimotor activation as in Equation (10). Spikes can be transmitted to this model via two custom synapse receptors, labeled *dynamic* and *static*. Because of the limitations of the ARM chips of the board, we had to implement fixed-point arithmetic functions for the implementation of Equation (8), such as division and exponentiation. The fiber length and speed can be injected into a running simulation by using a customized version of a spike live injector model found in the SpiNNaker library. In this way, information can be sent without stopping the simulation, fulfilling the real-time constraint of the whole system. To maximize the performance of the model and ensure real-time execution of the simulation, in every population of spindles, which should be relative to a single muscle, the fiber tension was computed for a single spindle unit, comprising all fiber types. Even if this decreases the accuracy of the simulation, this does not compromise the plausibility of the model, as it is equivalent to assuming that the central nervous system provides the same fusimotor stimulation for all spindles in a muscle. After the computations, the resulting Ia and II afferent rates were employed to generate spikes with Poisson inter-spike intervals from all spindle units. The random process was emulated using an approximation of a homogeneous Poisson process (Heeger, 2000):

(12) P{spike during δt}=rate·δt

where δ*t* is the duration of a simulation time step. This approach was suitable because we had fixed time bins (the simulation steps) and a small δ*t* (1 ms). At the end of the implementation procedure, the model, developed in C, could be instantiated from the SpiNNaker PyNN frontend (Davison et al., 2008). As for the NEST implementation, we had to add a Boolean flag to switch between primary and secondary afferent activity. In this case, developing two different models for primary and secondary activity would have a negative impact, as SpiNNaker allows us to simulate only homogeneous populations on its cores. This implies that primary and secondary afferents spindle models, relative to the same muscle, would be split into two populations, thus duplicating the fiber tension computation and forcing the user to create additional customized spike injectors.

## 3. Results

To assess the effectiveness of the proposed model, we performed several experimental trials. In particular, we validated the NEST and SpiNNaker implementations against a MATLAB Simulink simulation, comparable with the one performed in Mileusnic et al. (2006). Then, we employed the model to translate sensory information from different simulated and physical robotics systems, proving the generality of the approach. In all the experiments, we did not change the parameter values from the one reported in Mileusnic et al. (2006), which are optimized on cat soleous muscle recordings. However, the same anatomical structure exists in all mammals; therefore, because of the accurate representation of the anatomy of the model by changing the parameter values one could in principle reproduce the different properties of the various muscles. In our implementations, we left open the possibility to change such parameters by providing the appropriate NEST and PyNN interfaces.

### 3.1. Validation

As the first step, we developed a MATLAB Simulink model of Equations (1)–(11), whose results were directly, albeit empirically, comparable with those reported in Mileusnic et al. (2006). Then, we compared the results, in terms of afferent rates, of the two different implementations, with the Simulink reference by executing the same tasks in terms of fiber stretch and fusimotor stimulations. After the execution, the spike trains were recorded, and the rate was computed by sorting them into bins of fixed time intervals (30 ms) and counting them to compute the average rate for the bin.

In particular, we simulated a simple stretch, with the fiber length remaining constant at 0.95*L*_0_ for 1.1 s, stretching at 0.11*L*_0_/s for 1.1 s, and then remaining constant at 1.08*L*_0_ for the final 1.1 s. The same stretch was repeated under different fusimotor drives (γ_*dynamic*_ = 70 spikes/s and γ_*static*_ = 70 spikes/s). To provide the fusimotor stimulations in NEST and SpiNNaker simulations, we employed existing Poisson spike generators connected to the appropriate synapses types. Conversely, in the Simulink implementation, activities of γ-motoneurons were simulated by generating uniformly distributed spike trains. The number of muscle spindles simulated on NEST was 200, resulting in 400 simulated nodes, and 100 on SpiNNaker (200 nodes) because of memory per core limitations.

**Figure 3:**
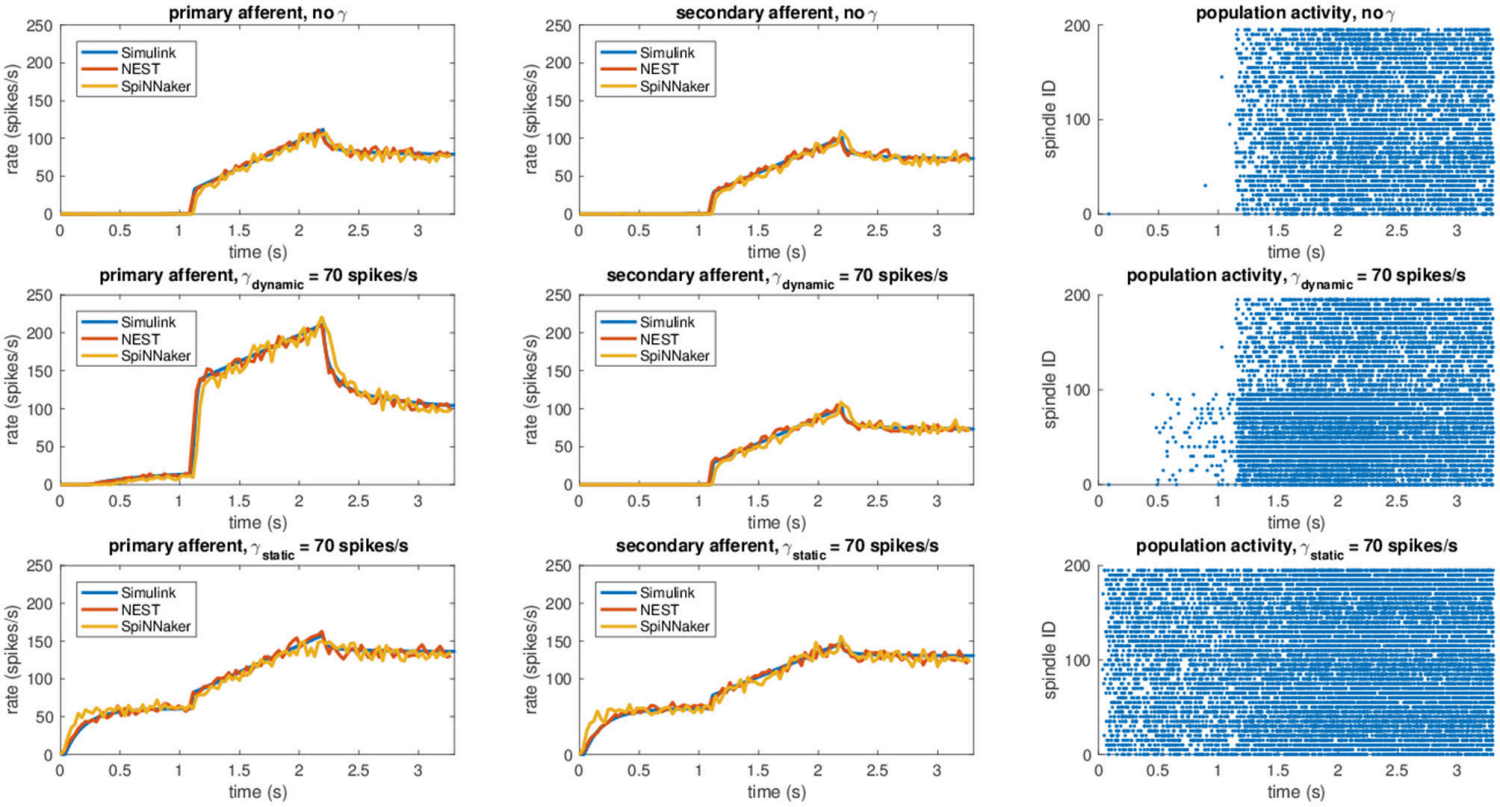
Comparison between the different implementations (Simulink, NEST, and SpiNNaker) for a stretching task with different fusimotor activities (no activity, dynamic at 70 spikes/s, and static at 70 spikes/s). The rows correspond to the different fusimotor activations. The first column shows the Ia afferent activity, in terms of spike rates, the second II afferent activity, and the third the raster plots of the neural population relative to the task, as produced by SpiNNaker. Spindles 0–99 simulate Ia activity, while spindles 100–199 simulate II activity. To improve visibility in the raster plots, only activity of 20% of the units of the spindle populations is displayed.

Results for this validation procedure can be found in Figure 3, where the response of the muscle spindle models, in terms of both computed spike rates and spike trains are shown. When no fusimotor stimulation is present, the spindle activity is null when the muscle is contracted; then, it starts to increase when the stretch begins and continues to rise as the stretch continues, and finally, it decreases and stabilizes at a certain level. Because there is no fusimotor stimulation, the activity of primary and secondary afferents is very similar. However, under a γ_*dynamic*_ stimulation of 70 spikes/s, Ia and II activities are radically different. In fact, primary afferents, the only ones affected by dynamic γ-motoneurons, have a greatly increased response, especially during the elongation phase, showing an increased sensibility to stretch speed. In contrast, static γ-motoneurons provide an overall increase in sensitivity of both Ia and II afferents, providing sensory feedback even when the muscle is contracted, as shown for γ_*static*_ = 70 spikes/s. From the results, we can confirm that the effects of γ-motoneuron stimulation during the simulations are in agreement with the spindle anatomy. Moreover, the results, in terms of computed spike rate, of the NEST and SpiNNaker implementations are very similar to the Simulink implementation, even if they are more noisy, thus proving their correctness. Finally, by comparing these results with those presented in Mileusnic et al. (2006) for a similar trial, we can observe that the removal of the second-order terms and the spike integration did not change the results significantly, as the behaviors of the firing rate responses are preserved.

The mean simulation time for the NEST implementation was 7.51 s on an i7-2760QM processor, implying a real-time factor of 0.44. The low real-time factor is due to the fact that the simulation must be stopped and restarted to set the values of *L* and $\dot{L}$. To compare the performance of the NEST model with those of other commonly used neural models, we performed a continuous simulation of 100 primary afferents under constant dynamic fusimotor stimulation of 100 spikes/s for 1 s, with *L* = 1 and $\dot{L}=0$, which resulted in an execution time of 0.48 s. By comparison, under the same stimulation, 100 leaky integrate and fire models had an execution time of 0.06 s and 100 adaptive leaky integrate and fire models run for 0.81 s. Therefore, the execution time of the NEST implementation falls between those of commonly used neural models. In contrast, simulations on SpiNNaker were able to run in real time, and 200 spindle models (primary and secondary) could be simulated by a single core, using only 1% of the maximum theoretical capabilities of the hardware.

### 3.2. Sensory translation experiments

Once validated, the model can be used to translate proprioceptive feedback from robotic systems, both simulated and physical. In our tests, we employed it to convert information coming from motor encoders into afferent activity, but the same mechanism can be applied to translate proprioceptive information from more realistic biomechanical models, such as musculoskeletal simulations, or different sensors, such as physical stretch sensors.

**Figure 4:**
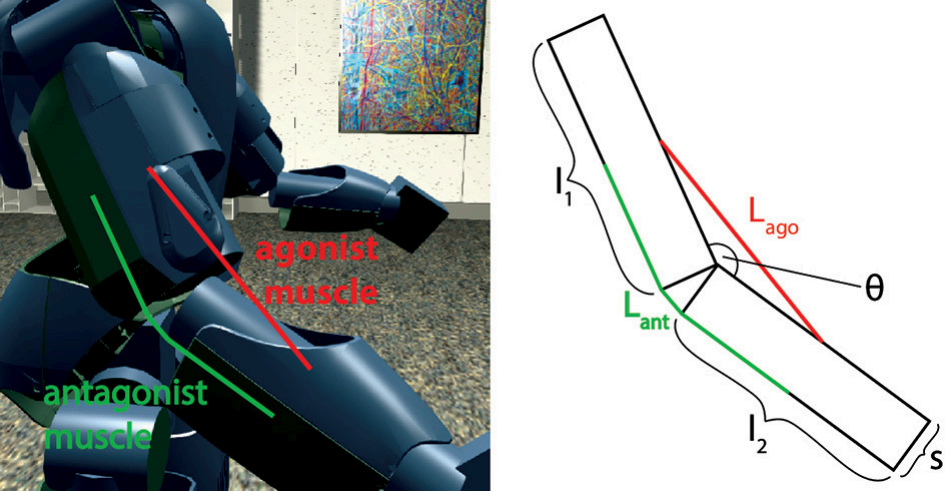
Simulated agonist-antagonist muscle pair for a two-link system and its application to the elbow joint of the iCub robot (left). The length of the two muscles can be expressed as a function of the kinematic parameters of arm and forearm and of the current joint angle.

First, the model was tested by embedding the NEST implementation in the Neurorobotics Platform, a simulation tool that is able to coordinate physical and neural simulations to create neurorobotic action-perception closed loops (Falotico et al., 2017). This platform allows the user to easily transfer data between the two simulations by implementing transfer functions that convert data coming from one simulation into suitable inputs for the other (Hinkel et al., 2017). In our case, we had to integrate the NEST spindle model in the list of possible devices and then develop the transfer functions for the specific setups. In particular, we connected the spindle neural models to two different robotic embodiments: an iCub robot (Metta et al., 2008) and a simulated mouse body. In principle, every joint connecting two links can be considered actuated by an agonist-antagonist pair of muscles. Therefore, sensory information should be translated in terms of stretches of such muscles. We demonstrate this on a simulated iCub robot, where we employed the spindle model to translate information received from the elbow encoder into afferent activities for an antagonistic pair of simulated muscles. The stretch and speed of the simulated muscles were computed geometrically, as a function of the encoder values ($\theta \left(t\right),\dot{\theta }\left(t\right)$) and of kinematic parameters of the links (arm and forearm, cf. Figure 4). Assuming, for simplicity, that the muscles are attached in the middle point of the two links and that 0 ≤ θ(*t*) ≤ 2π, the stretch and speed for the agonist and antagonist muscles can be computed as follows:

(13) $$L_{ago}(t)=\sqrt{\frac{l_{1}^{2}}{4}+\frac{l_{2}^{2}}{4}-\frac{l_{1}l_{2}}{2}\cos(\theta (t))}$$

(14) $$\dot{L_{ago}}(t)=\frac{\frac{l_{1}l_{2}}{4}\sin(\theta (t))\cdot \dot{\theta }(t)}{L_{ago}(t)}$$

(15) $$L_{ant}(t)=\frac{l_{1}}{2}+\frac{l_{2}}{2}+2s\cdot \sin\left(\frac{\pi -\theta (t)}{2}\right)$$

(16) $$\dot{L_{ant}}(t)=-2s\cdot \cos\left(\frac{\pi -\theta (t)}{2}\right)\;\cdot \;\dot{\theta }(t)$$

This system of equations can be employed on any two-link, asymmetric system (e.g., thigh-leg), by simply changing the kinematic parameters of the links or the attachment point of the two muscles.

**Figure 5:**
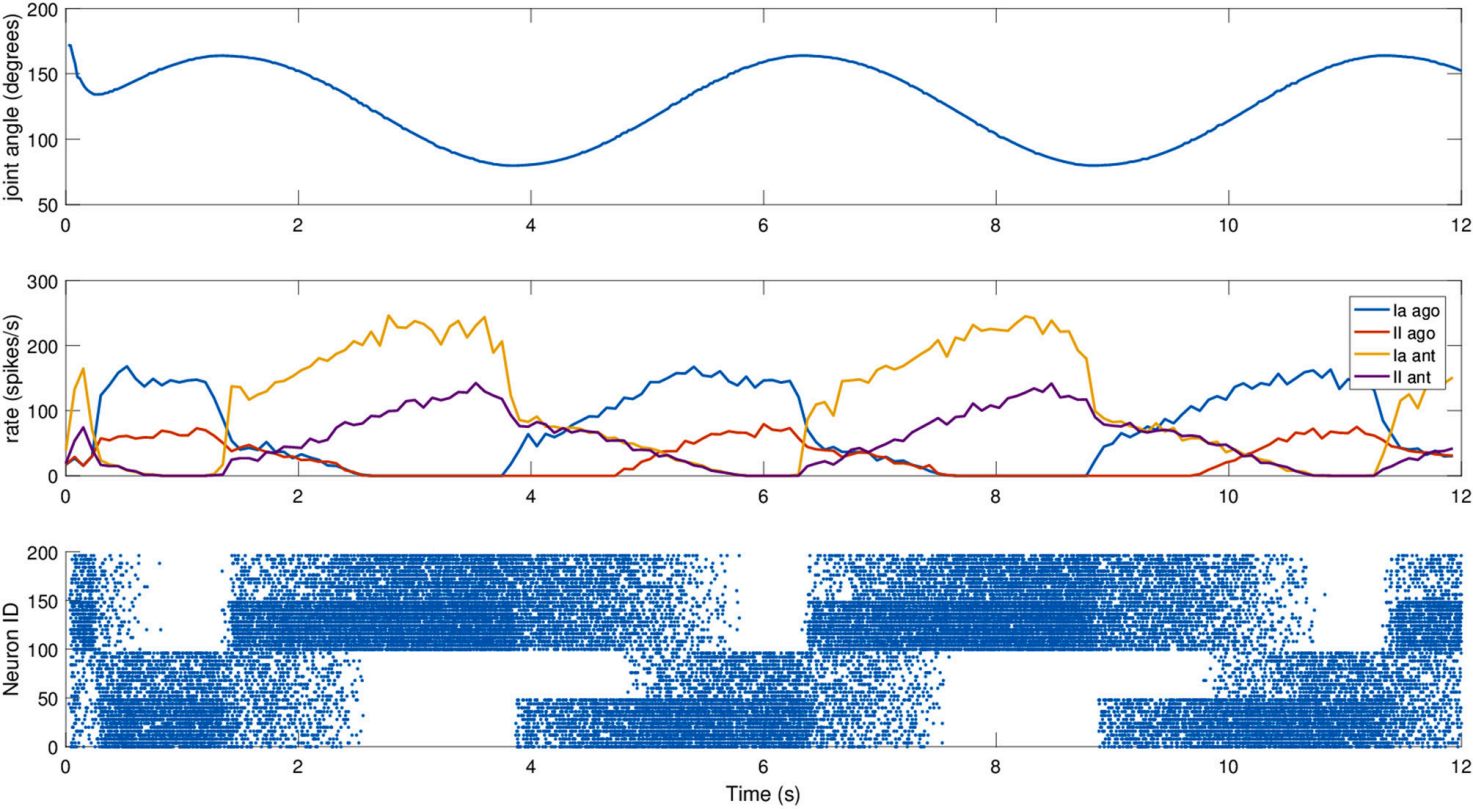
Afferent activity for the agonist-antagonist pair during a sinusoidal movement of the iCub elbow (top). Computed rates for Ia and II activities are shown in the middle, and the raster plot for the two muscle populations is on the bottom. To improve visibility in the raster plot, only activity of 25% of the units of the spindle populations is displayed.

To test it, a simulated iCub robot was placed inside a virtual room, where the experiment took place. A sinusoidal motion with a peak-to-peak amplitude of 45 degrees and a frequency of 0.2 Hz was given to the elbow motor to simulate a rhythmic co-activation of the two muscles and an alternation of stretching and elongating of the corresponding spindles. The motion was centered on what we considered the resting position for the computation of *L*_0_, 125 degrees, an angle where none of the two simulated muscles is completely stretched (Figure 4). To stress the difference between primary and secondary afferent endings, fusimotor activation was set to γ_*dynamic*_ = 70 spikes/s during the trial. The activity of the spindles during this trial are shown in Figure 5. It can be observed that the activity is not symmetric, as expected from the geometrical translation model employed, but that afferent rates values correctly alternate between the two simulated muscles, following the elbow motion. Moreover, Ia activity is higher than II, thanks to the dynamic fusimotor drive. Because no static fusimotor activity is present, when the fibers are contracted, there is no activity to be recorded. The computational burden of the simulation of 200 spindle models, combined with the physical simulation, resulted in a real-time factor of the whole coordinated simulations of 0.16.

**Figure 6:**
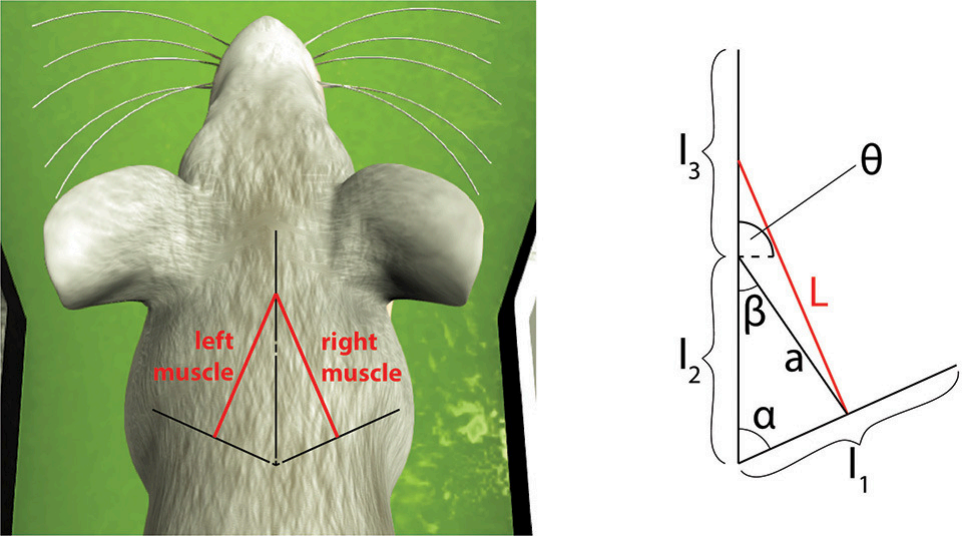
Simulated muscles for a symmetric three-link system and its application to the virtual mouse neck. The length of the muscle can be expressed as a function of the kinematic parameters of the three links and of the current two joint angles between them.

To consider a different link system, we connected the spindle model to simulate the afferent activities of muscles connected to a three-link kinematic chain, the shoulder-neck-head link system of a simulated mouse body, inside the Neurobotics Platform. The model consists of a rigid skeleton actuated by rotational joints, covered with deformable skin. The subset of interest of the skeletal model can be seen in Figure 6, where the relevant kinematic parameters are shown.

Compared to the previous case, this link system is symmetric, so the same equations can be used to compute *L*(*t*) and $\dot{L}\left(t\right)$ by just changing the sign of θ(*t*) and $\dot{\theta }\left(t\right)$. Moreover, in this case, the muscle is attached to the first and third links. To simplify the equations, we assumed the angle between the first two links (α) to be constant. However, small modifications are needed to consider it variable in time. The length and speed of the muscle spindles can be computed using the following equations:

(17) $$a=\sqrt{\frac{l_{1}^{2}}{2}+l_{2}^{2}-l_{1}\cdot l_{2}\cdot \cos(\alpha )}$$

(18) $$\beta =\arccos\left(\frac{a^{2}+l_{2}^{2}-{\left(\frac{l_{1}}{2}\right)}^{2}}{2\cdot a\cdot l_{2}}\right)$$

(19) $$L(t)=\sqrt{\frac{l_{3}^{2}}{2}+a^{2}-a\cdot l_{3}\cdot \cos\left(\theta (t)+\frac{\pi }{2}-\beta \right)}$$

(20) $$\dot{L}(t)=\frac{\frac{a\cdot l_{3}}{2}\sin\left(\theta (t)+\frac{\pi }{2}-\beta \right)\cdot \dot{\theta }(t)}{L(t)}$$

**Figure 7:**
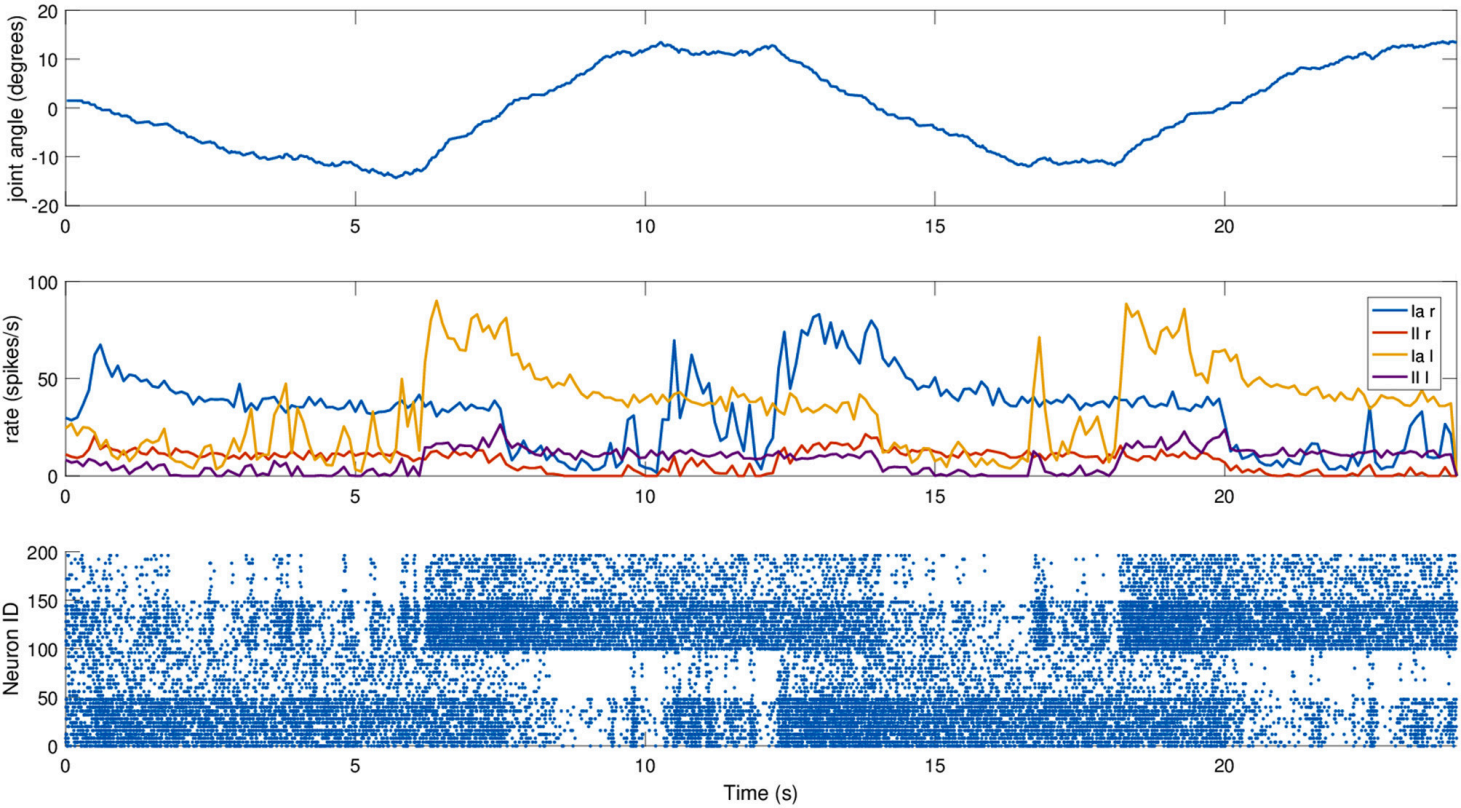
Afferent activity for the neck muscle pair during a screen viewing experiment. The head motion during the trial is on top, computed rates for Ia and II activities are shown in the middle, and the raster plot for the two muscle populations is on the bottom. To improve visibility in the raster plot, only activity of 25% of the units of the spindle populations is displayed.

For this experiment, we did not move the mouse head directly, but relied on an existing setup where the mouse moved its head as part of a Braitenberg-like experiment: the mouse is placed in a Y-maze with two displays placed at the end of the corridors, and the mouse should look at the red one, away from the blue one, using a trivial neural model. The two screens switched states every 6 s, and the motion relies only on visual information, particularly on the percentage of red pixels in the camera image. The fact that the relevant joint is not explicitly controlled provides a more realistic scenario. We recorded the spindle activities during such a trial, again providing dynamic fusimotor drive (γ_*dynamic*_ = 70 spikes/s) to enhance the differences between Ia and II activity. Results for the experiment can be found in Figure 7, where it can be observed that the activities of the spindles of the two muscles are symmetric and out of phase, in correspondence with the head motion. The controller does not provide smooth movements of the head, so the activity of the spindle models is noisy, especially in the presence of momentary and sudden changes in the fiber stretch speed. However, the activity of the two muscles and of the two different afferent types are clearly distinguishable from one another. It can be noticed that, because of the low range of motion, the firing rate of II afferents is very low. In addition, the stretching speeds are very low, but rates of the Ia afferents are still high thanks to the dynamic fusimotor activity. Finally, activity for contracted spindles is close to null, except for activity generated by the noisy input. In this case, the real-time factor of the coordinated simulation was 0.17.

**Figure 8:**
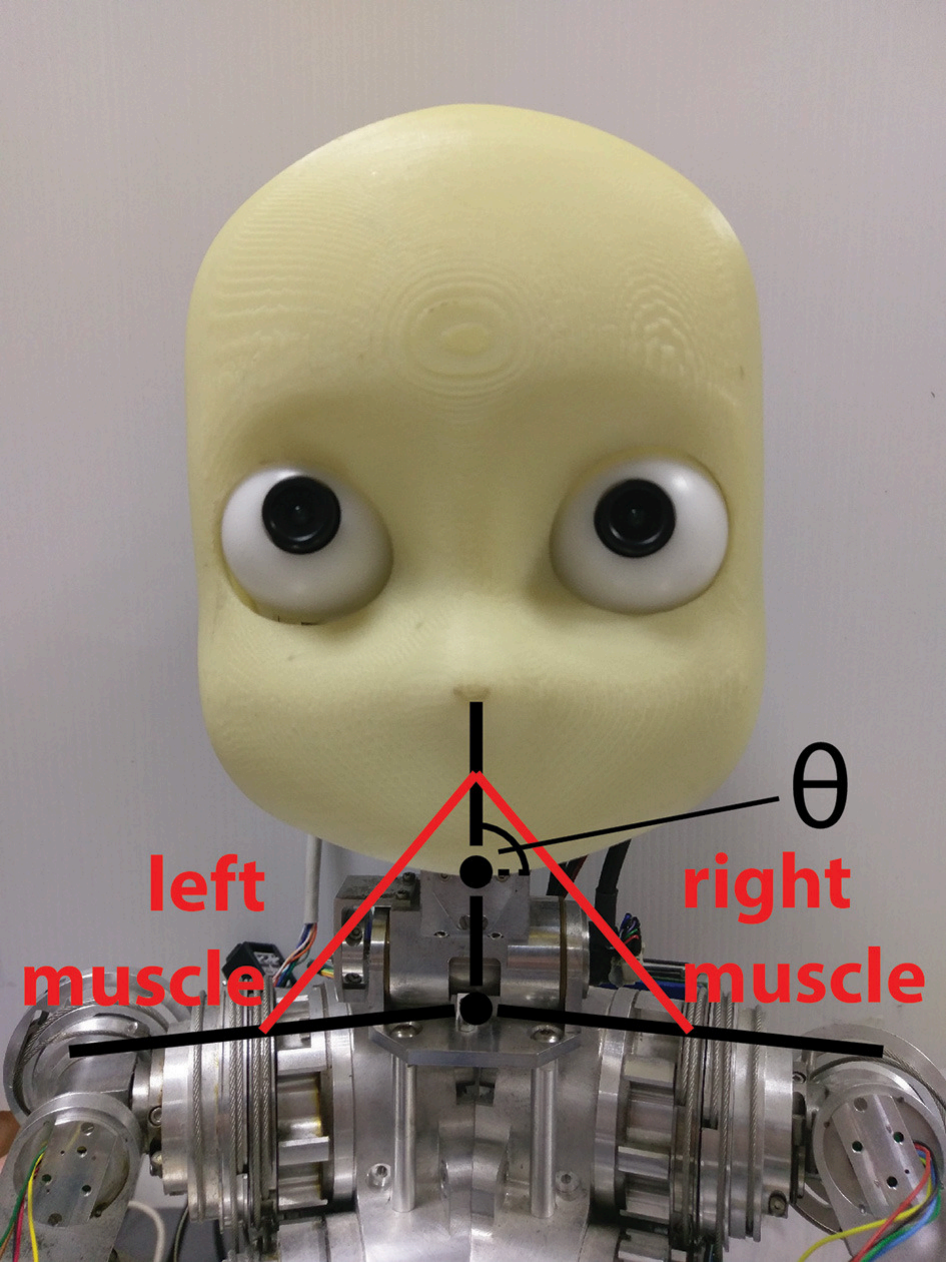
Simulated muscles for the iCub neck roll using the three-link system previously described for the mouse head.

To show the real-time capabilities of the SpiNNaker implementation, we employed the spindle model on a physical robotic platform. We considered a three-link system starting from the shoulder link up to the head of an iCub robot, actuated by the neck roll joint. Because the link system has the same structure as the previous case, we could translate encoder values for such a joint using Equations (17)–(20) by changing the kinematic parameters to match the iCub kinematics (Figure 8).

**Figure 9:**
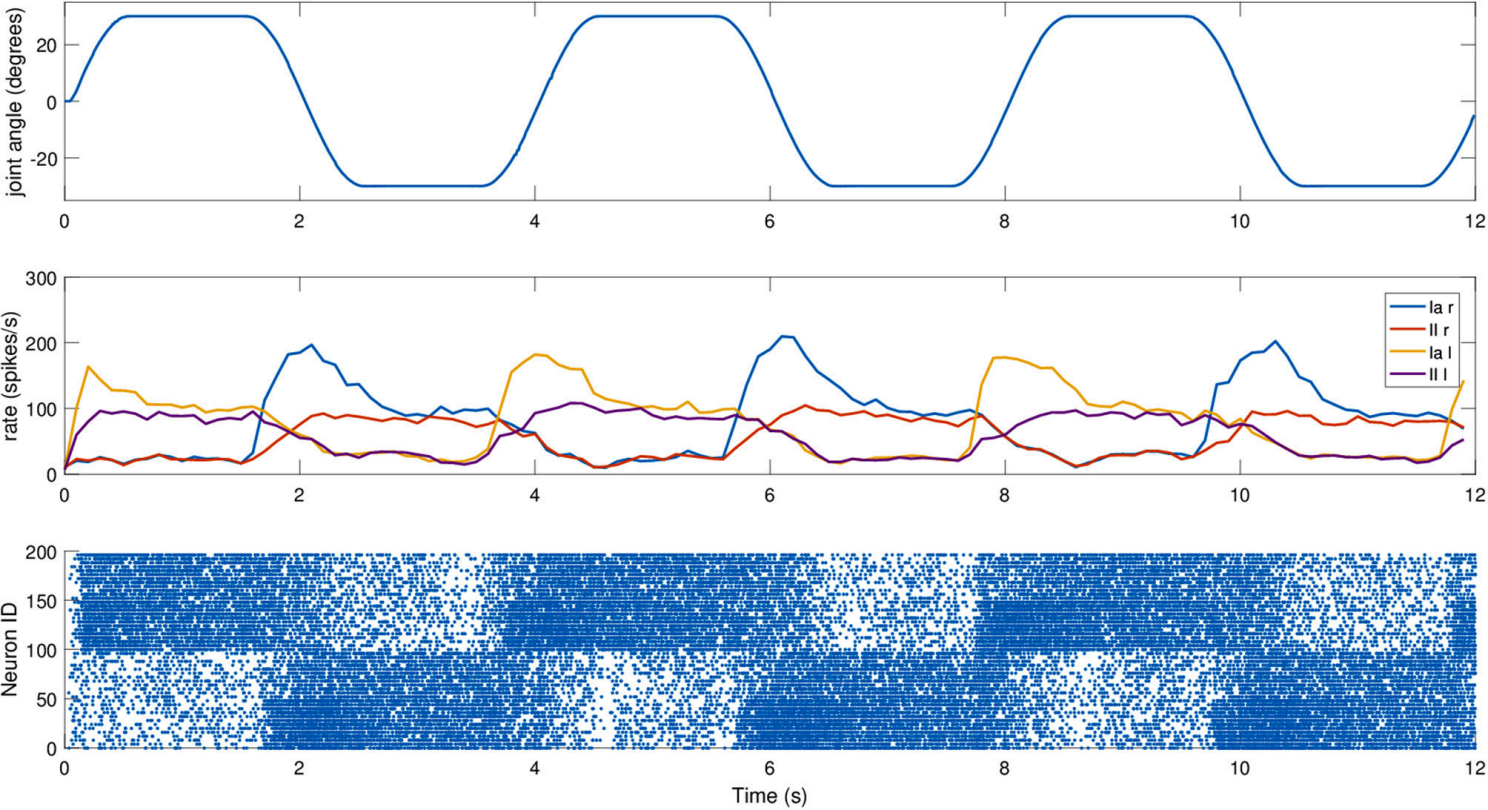
Afferent activity for the neck muscle pair simulated on the iCub robotic platform. The head motion during the trial is on top, computed rates for Ia and II activities are shown in the middle, and the raster plot for the two muscle populations is on the bottom. To improve visibility in the raster plot, only activity of 25% of the units of the spindle populations is displayed.

To actually send the translated encoder values to SpiNNaker, as well as to retrieve live spiking activity from the simulation, a proper real-time data exchange middleware was developed in C++. The joint speed cannot be directly retrieved from the motor, so it was necessary to compute it. To test the robustness of the spindle model against input noise, a simple single-step differentiation was employed. The neck roll joint was then moved in a sinusoidal fashion, with a peak-to-peak amplitude of 30 degrees and a frequency of 0.5Hz, but the maximum stretch was maintained for 1 s on every side, by keeping the head still when it reached the maximum range of motion, resulting in a motion with a period of 4 s. During this trial, fusimotor activity was kept at γ_*dynamic*_ = 80 spikes/s and γ_*static*_ = 40 spikes/s. Results for this trial can be found in Figure 9. As expected, the activities of the two sets of spindles are symmetric, out of phase from each other, and in sync with the motion. It is interesting to notice how Ia and II activities for the same muscle differ only during the actual motion part, while they tend to be almost the same when the head is still. Therefore, a model of the central nervous system, by properly activating γ-motoneurons, could really be able to discriminate between motion and different stretch levels. Moreover, thanks to static fusimotor activation, the spindles are overall more sensitive, and even when they are contracted, some activity is present. For the simulation of this model, to simulate the 200 spindles, divided into two neural populations, we used two cores of the SpiNN-5 board and thus only 0.2% of its maximum theoretical capabilities.

## 4. Conclusion

In this work, we presented a spike-based proprioceptive feedback transmission mechanism able to produce biologically realistic firing activity, as produced by muscle spindles, that can be fully integrated in spiking neural network simulation and on neuromorphic hardware. The mechanism, which emulates the dynamics of muscle spindles under stretch and γ-motoneurons activation, was implemented on two different simulators: NEST, a commonly employed spiking neural network simulator, and SpiNNaker, a neuromorphic hardware platform. The model was obtained by modifying an existing muscle spindle model (Mileusnic et al., 2006) to perform spike integration to compute the fusimotor drives, to produce a spiking output, and to simplify the computation. The results show that the proposed implementations are accurate, with respect to a non-spiking implementation. The transmission mechanism is flexible enough to be employed on different embodiments, both simulated and physical.

This work can be beneficial for robotics as well as neuroscience. With respect to robotics, the model is naturally conceived to reproduce the sensory feedback of muscoloskeletal bodies but can be adapted to motor-actuated robots. In particular, we employed it in two simulation scenarios, with two different simulated robots and with a physical robot to transmit sensory information from motor encoders. By doing so, we actually created a general method of conversion between motor encoders and muscle lengths for kinematic structures with two and three sequential links. The sensory information translated by the spindle models can be used to create biologically inspired brain-like controllers, something that cannot be achieved with tailor-made translations, like the one proposed in Bouganis and Shanahan (2010), which can only work for specific tasks. The neuromorphic implementation guarantees real-time performance and scalability for real robotic applications. Most humanoid robots have no more than 50 degrees of freedom, and if we consider all of them as actuated by a pair of muscles and that the population relative to one muscle can be simulated on a single SpiNNaker core, the whole simulation of the afferent fibers for all muscles will not occupy more than 12% of the processing capability of a SpiNN-5 board.

From a neuroscientific point of view, the model can be used to further explore action-perception loops for reflexes and voluntary movements. One of the possible extensions of the proposed model can be based on the integration of the modulation of different responses by γ-drive. In a previous work Grandjean and Maier (2014), supported by experimental data from a cat during passive sinusoidal stretches, the authors proposed a computational model able to predict type Ia and IIa muscle spindle activity as a function of the time-varying γ-drive. The proposed model is also fit to be a testbed for such investigations, as it can be simply coupled with complex biomechanical models and integrated in large-scale NEST simulations. Moreover, being spike-based, it could also help to simulate and reproduce detailed spike data, such as data recorded from the spinal cord with microelectrode arrays (Arle et al., 2012).

The proposed model and its implementations have some limitations. While the simplified differential equation does not introduce substantial effects, the spike integration mechanism tends to be less accurate, with respect to the original Hill-type function, under low (< 30 spikes/s) and high (> 150 spikes/s) fusimotor activity, either underestimating it or overestimating it. Regarding the implementations, the NEST model cannot be run continuously because the muscle length and stretch speeds can only be set while the simulation is paused. While pausing and restarting may not be an issue in many synchronized closed-loop applications such as the one presented in this work, it actually slows down the overall neural simulation. Possible solutions to overcome this problem include modifying the implementation to make enable it to receive data through a socket or employing synchronization frameworks capable of injecting information while the simulation is running, such as MUSIC (Djurfeldt et al., 2010). The SpiNNaker implementation is limited in the accuracy of the computation because of the fixed-point arithmetic and in the number of models that can be simulated on a single core. This could force the user to split the population relative to a muscle if a biologically accurate number of spindles is employed, with a consequent duplication of the code for the live injection of length and stretch information.

In the future, we plan to employ this proprioceptive feedback mechanism, alongside similar spike-based and neuromorphic translational models for other sensors and actuators, for the creation of complete action-perception loops for both neuroscientific and robotic applications.

## Author contributions

The proprioceptive feedback model was developed, implemented, and tested by LV and EF. LV, EF, and CL wrote and reviewed the manuscript.

### Conflict of interest statement

The authors declare that the research was conducted in the absence of any commercial or financial relationships that could be construed as a potential conflict of interest.
